# PP62 Improving Patients’ Access To Medicines Through Health Technology Assessment In Ukraine: Measuring The Impact Of The National HTA Roadmap

**DOI:** 10.1017/S0266462324002332

**Published:** 2025-01-07

**Authors:** Oresta Piniazhko, Marharyta Khmelovska, Valeriia Serediuk, Mykhaylo Babenko, Mykhailo Lobas, Kostyantyn Kosyachenko, Rabia Sucu, Nataliia Valuieva

## Abstract

**Introduction:**

In 2020, health technology assessment (HTA) became mandatory for any new medicine covered by public funds. The aim of the review is to present the impact of HTA implementation despite Russia’s full-scale war against Ukraine. Thanks to the introduced reforms, including HTA introduction, the coverage from the public payer in providing patients with medicines increased from 11 percent in 2013 to 34 percent in 2023.

**Methods:**

This review summarizes all Department of HTA conclusions with recommendations made during 2023, decisions made by the Ministry of Health (MOH) based on these recommendations and their impact on patient access, and the steps made to further institutionalize HTA in Ukraine. In 2023, the HTA guidelines for medicines were revised based on the lessons learned and global experience along with knowledge exchange with the United Kingdom HTA agency, NICE International.

**Results:**

In 2023, the HTA Department prepared 19 HTA conclusions. The majority concerned treatment of oncologic diseases (32%), orphan diseases (21%), and multiple sclerosis (16%). HTA assessments resulted in recommendations for listing (84% cases). Fifty-three percent of medicines were recommended for managed entry agreement, and the MOH decided to forward 10 medicines for negotiations in 2023. Thirty-two percent of recommendations considered inclusion into the National Essential Medicines List (NEML); 16% with further access through the reimbursement program. Almost 70 percent of total need in NEML is currently covered by public funds, compared to 40 percent in 2020 (according to the Support in Market Development (SMD) analytical database).

**Conclusions:**

The HTA function in Ukraine has evolved since 2020 with a visible impact on patients’ access. Introduction of new procedures, such as HTA for medical devices, may improve patient access to health technologies and provide evidence for rational and transparent decision-making processes, with gradual transformation of the Department of HTA at the State Expert Centre into an independent HTA agency by 2026.

